# Supplementary material for: Oral Contraceptive Use and Reproductive History in Relation to Metabolic Syndrome Among Women from KNHANES 2010–2023
Source: J Clin Med. 2025 Sep 7;14(17):6319. doi: 10.3390/jcm14176319 (PMC12429387; doi:10.3390/jcm14176319)
Supplement: Supplementary file 1 [file jcm-14-06319-s001.zip › jcm-3832224-supplementary.pdf]

Supplementary Table S1. Missing analysis of covariates.

Variables	n	missing	missing(%)
BMI	31090	88	0.28
Age	31178	0	0.00
Income level	31041	137	0.44
Education level	31146	32	0.10
Spouse	29376	1802	5.78
Smoking Status	31121	57	0.18
Drinking habit	31092	86	0.28
Walking per week	31168	10	0.03
Exercise per week	31175	3	0.01
Waist circumference	30948	230	0.74
Blood pressure	31057	121	0.39
High-density lipoprotein	30261	917	2.94
Triglycerides	30436	742	2.38
Fasting glucose	31178	0	0.00
Metabolic syndrome	29984	1194	3.83
Oral contraceptives	31178	0	0.00

Supplementary Table S2 Univariate analysis of risk factors for metabolic syndrome by menopa use status.

Variables	Pre Menopause Crude OR (95% CI)	Post Menopause Crude OR (95% CI)
**Age per 1 years**	1.10 (1.09–1.11)	1.10 (1.09–1.10)
**BMI per 1 kg/m^2**	1.43 (1.40–1.46)	1.33 (1.31–1.35)
**Education level, College**	1.00 (ref.)	1.00 (ref.)
High school	2.07 (1.83–2.33)	1.31 (1.16–1.49)
Middle school	4.53 (3.68–5.59)	2.00 (1.75–2.29)
Elementary	6.87 (5.47–8.63)	2.94 (2.60–3.34)
**Income level, Q5: High**	1.00 (ref.)	1.00 (ref.)
Q4: Middle-high	1.28 (1.07–1.52)	1.17 (1.03–1.32)
Q3: Middle	1.51 (1.26–1.80)	1.32 (1.16–1.49)
Q2: Low-middle	1.62 (1.36–1.94)	1.43 (1.27–1.62)
Q1: Low	2.10 (1.78–2.49)	1.58 (1.39–1.79)
**Spouse, Yes**	1.00 (ref.)	1.00 (ref.)

No	1.78 (1.48–2.13)	1.34 (1.22–1.48)
**Smoking status, Never smoker**	1.00 (ref.)	1.00 (ref.)
Ex-smoker	0.91 (0.74–1.12)	1.16 (0.95–1.41)
Current smoker	1.41 (1.15–1.72)	1.13 (0.93–1.37)
**Drinking habit, Never drinking**	1.00 (ref.)	1.00 (ref.)
Normal drinking	1.18 (1.01–1.37)	1.26 (1.08–1.46)
High drinking	2.55 (2.05–3.17)	1.64 (1.38–1.95)
**Walking per week, 0 day**	1.00 (ref.)	1.00 (ref.)
1-3 days	1.02 (0.91–1.14)	1.01 (0.92–1.11)
4-7 days	1.12 (0.96–1.31)	1.28 (1.16–1.42)
**Exercise per week, 0 day**	1.00 (ref.)	1.00 (ref.)
1-3 days	0.63 (0.53–0.76)	0.60 (0.53–0.68)
4-7 days	0.81 (0.63–1.03)	0.57 (0.49–0.66)
**Age of Menarche per 1 year**	1.09 (1.05–1.12)	1.07 (1.05–1.09)
**Pregnancy per 1 times**	1.18 (1.14–1.21)	1.10 (1.07–1.12)
**Age of First Delivery per 1 year**	0.92 (0.91–0.94)	0.94 (0.93–0.95)
**Breast Feeding per 1 child**	1.14 (1.09–1.18)	1.16 (1.14–1.19)
**Oral Contraceptives, Not used**	1.00 (ref.)	1.00 (ref.)
Used	1.42 (1.22–1.65)	1.42 (1.28–1.56)

Supplementary Table S3. Adjusted odds ratios for metabolic syndrome and its components ac cording to OC use (categorical variable, waist circumference > 80 cm), stratified by menopaus al status.

	Pre Menopause, aOR (95% CI)	Post-Menopause, aOR (95% CI)
**MetS (3+ of Risks)**	**1.35 (1.12–1.64)**	1.10 (0.98–1.23)
5' of Risks	1.03 (0.68–1.55)	1.01 (0.87–1.18)
4+ of Risks	**1.31 (1.01–1.70)**	1.06 (0.95–1.20)
2+ of Risks	1.10 (0.95–1.29)	1.09 (0.95–1.24)
1+ of Risks	0.90 (0.77–1.05)	1.04 (0.84–1.28)
High Blood Pressure	**1.20 (1.01-1.43)**	**1.30 (1.17-1.45)**
High Fasting Blood Sugar	1.03 (0.88-1.20)	0.95 (0.86-1.04)
High Triglycerides	**1.23 (1.04-1.44)**	1.02 (0.92-1.14)
Low HDL Cholesterol	0.99 (0.86-1.13)	1.05 (0.95-1.17)
Excess Waist	1.02 (0.82–1.26)	0.91 (0.78–1.06)
